# Supplementary figures and images for: RNA binding protein ZCCHC24 promotes tumorigenicity in triple-negative breast cancer
Source: EMBO Rep. 2024 Oct 17;25(12):12. doi: 10.1038/s44319-024-00282-8 (PMC11624195; doi:10.1038/s44319-024-00282-8)

**Hoechst****ZEB1****ZCCHC24****MERGE****Case1**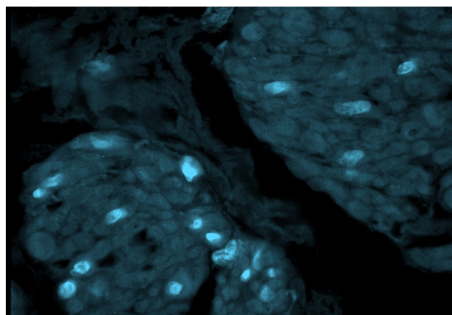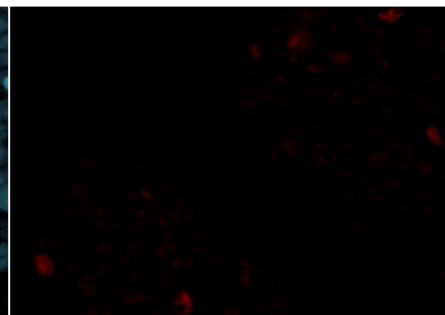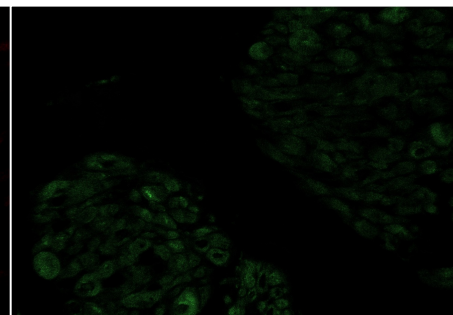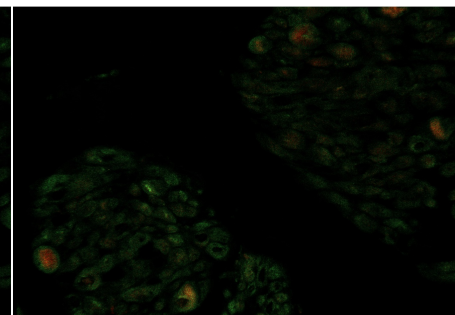**Case2**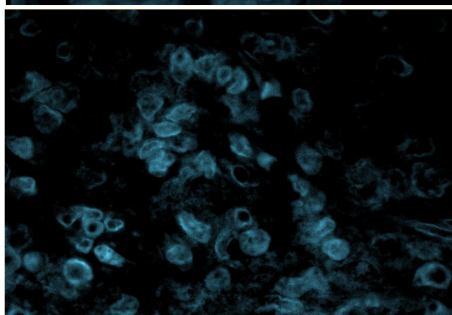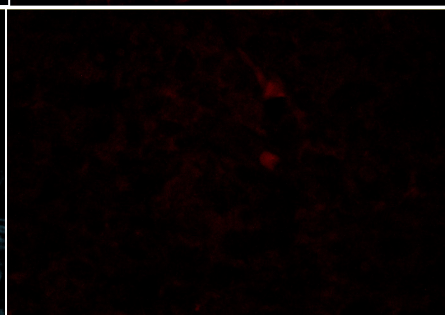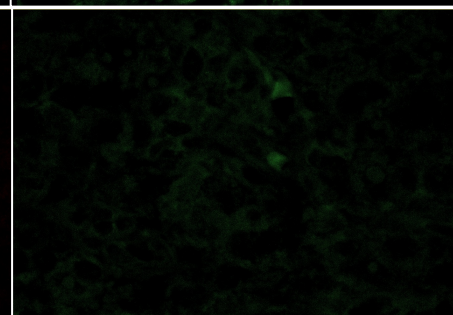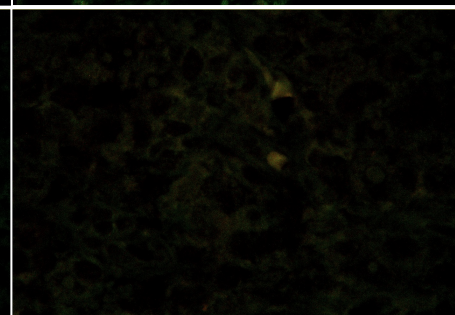**Case3**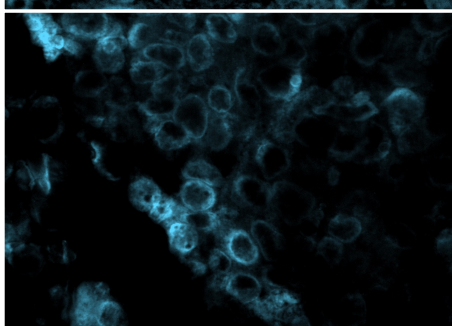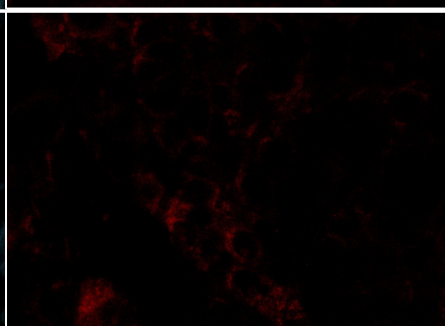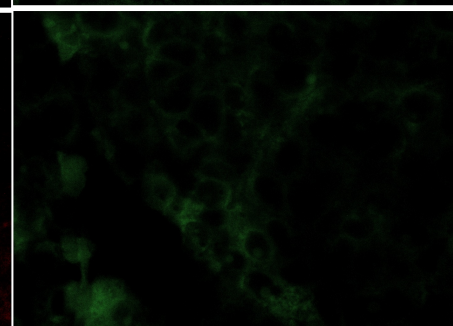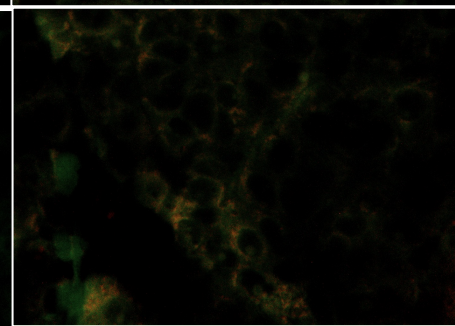**Case4**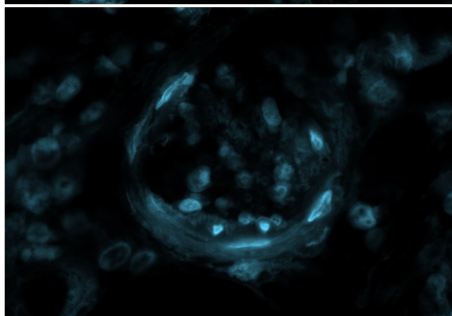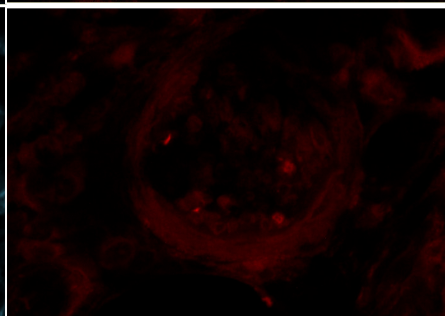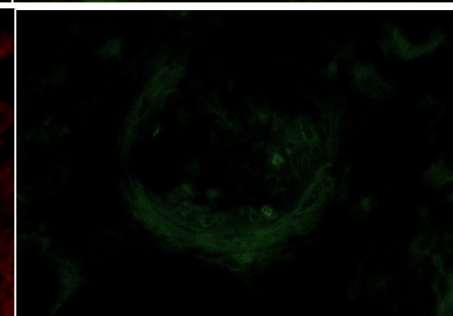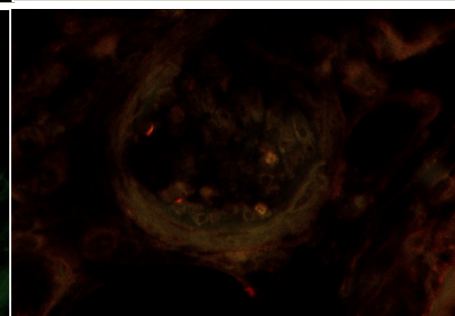

Supplement: Supplementary file 11 — Source data Fig. 4 [file 44319_2024_282_MOESM11_ESM.zip › Fig 4G Source.pdf]

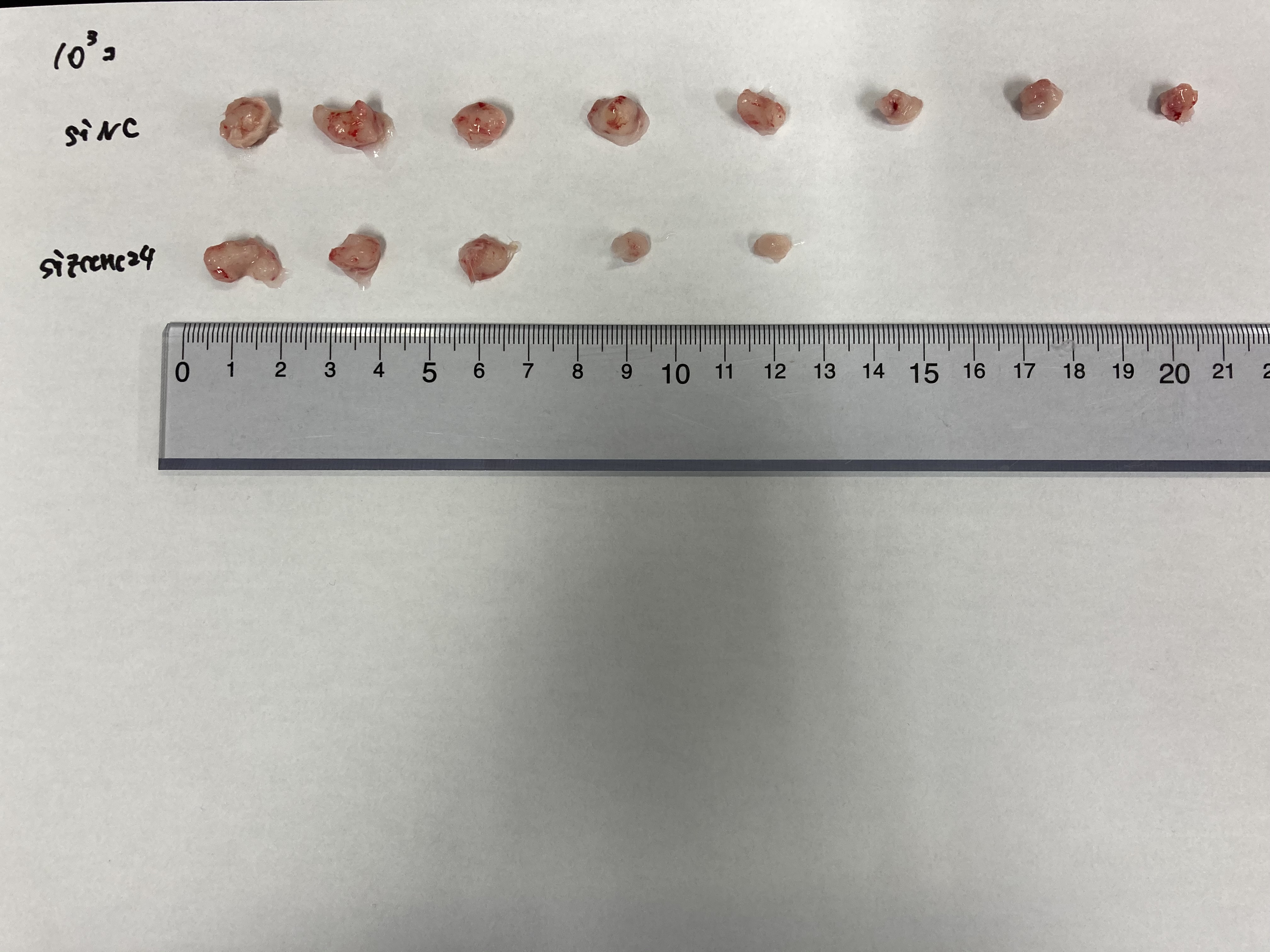

Supplement: Supplementary file 12 — Source data Fig. 5 [file 44319_2024_282_MOESM12_ESM.zip › Fig5B Source Image/Fig5B_1000.jpeg]

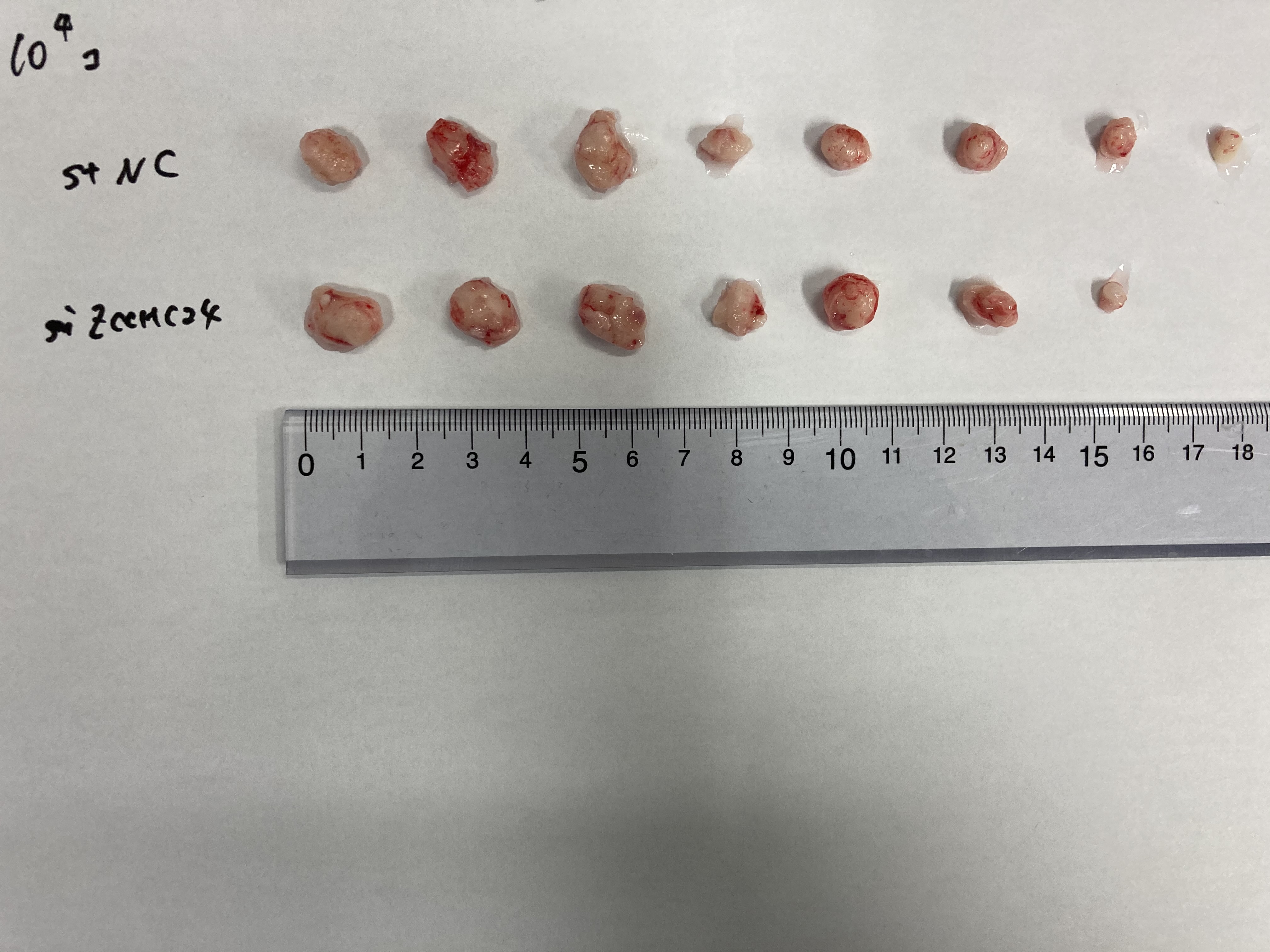

Supplement: Supplementary file 12 — Source data Fig. 5 [file 44319_2024_282_MOESM12_ESM.zip › Fig5B Source Image/Fig5B_10000.jpeg]

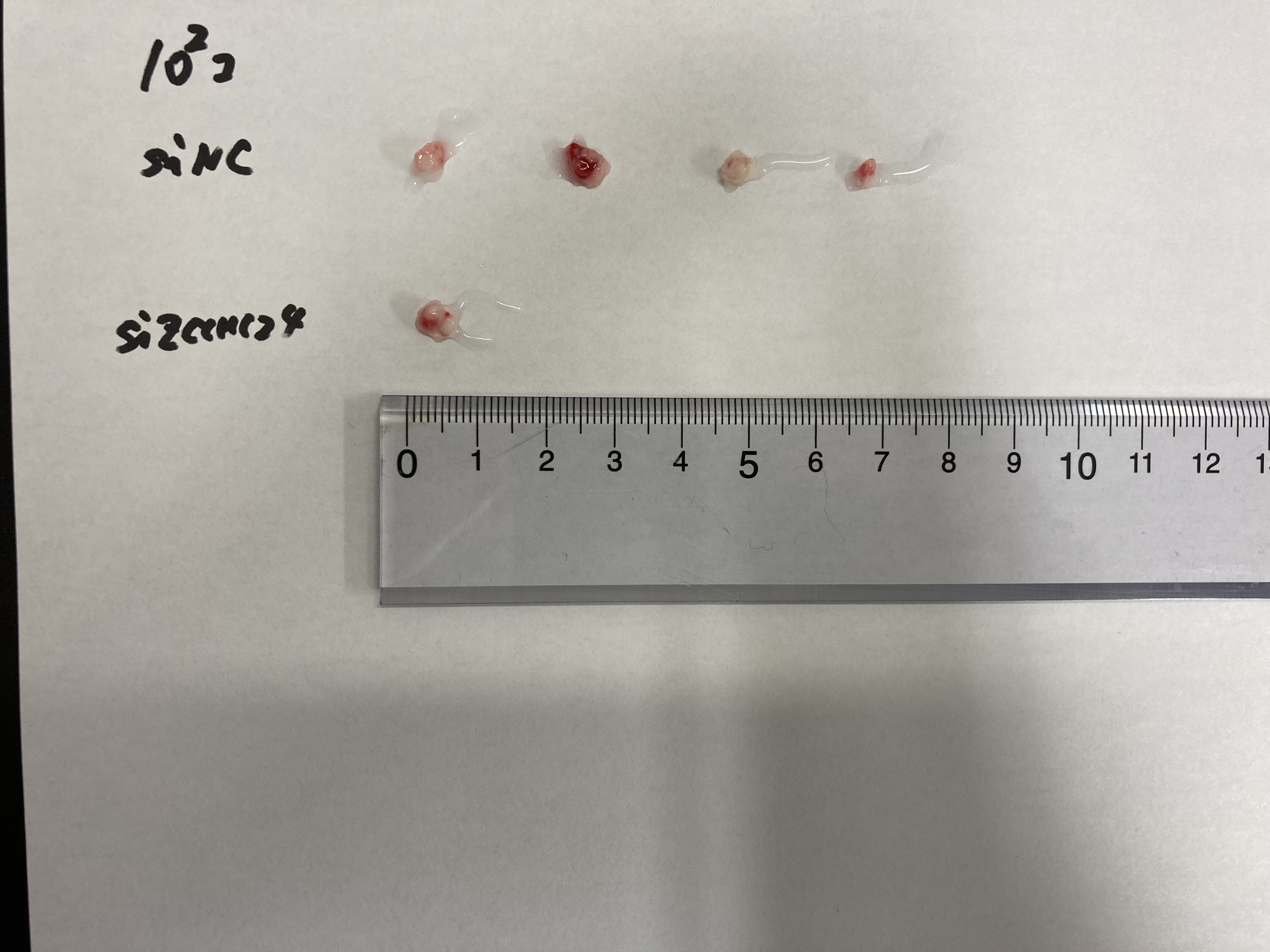

Supplement: Supplementary file 12 — Source data Fig. 5 [file 44319_2024_282_MOESM12_ESM.zip › Fig5B Source Image/Fig5B_100.jpeg]
