# Supplementary material for: RNA binding protein ZCCHC24 promotes tumorigenicity in triple-negative breast cancer
Source: EMBO Rep. 2024 Oct 17;25(12):12. doi: 10.1038/s44319-024-00282-8 (PMC11624195; doi:10.1038/s44319-024-00282-8)
Supplement: Supplementary file 14 — Expanded View Figures [file 44319_2024_282_MOESM14_ESM.pdf]

Expanded View Figures

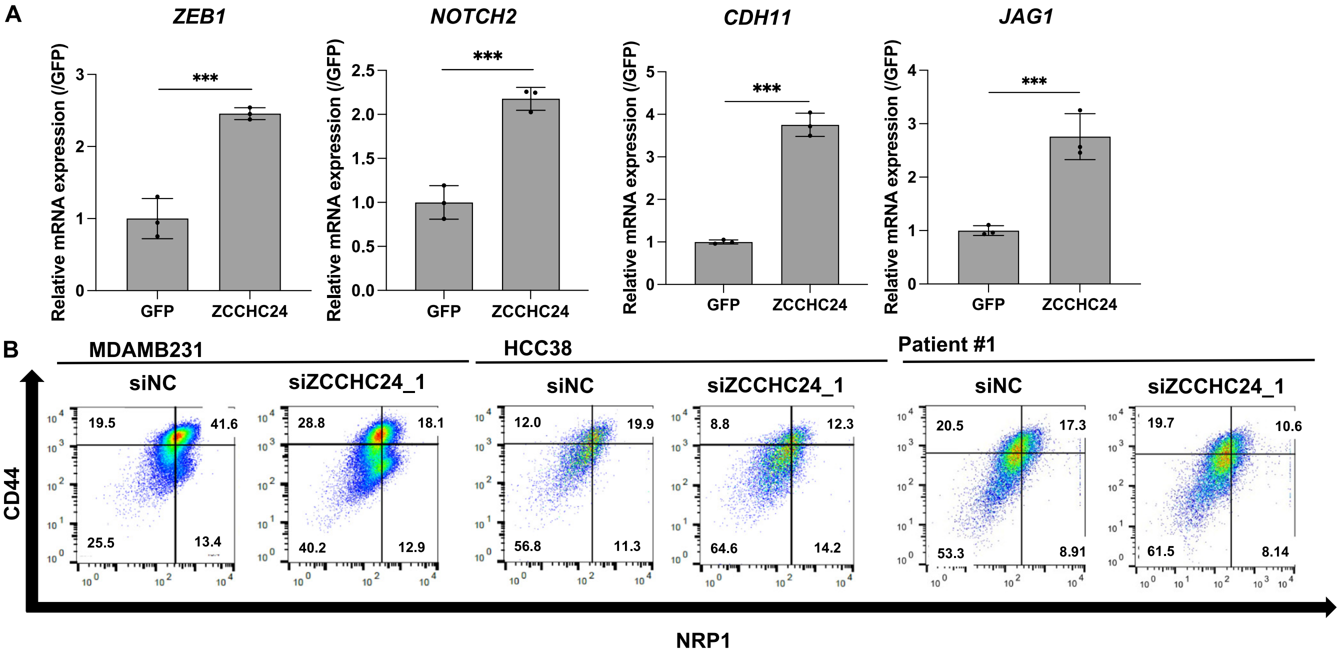

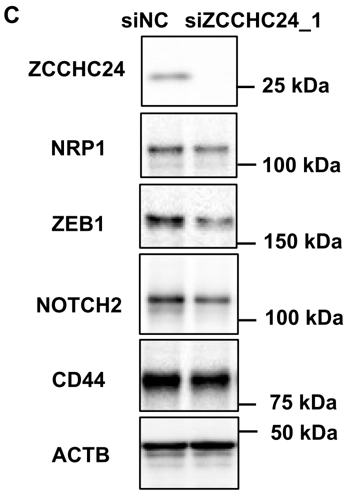

D

Term	*P*-value
negative regulation of apoptotic process	7.1.E-05
positive regulation of cardiac epithelial to mesenchymal transition	1.3.E-04
angiogenesis	1.3.E-04
maintenance of blood-brain barrier	4.3.E-04
positive regulation of peptidyl-tyrosine phosphorylation	5.0.E-04
negative regulation of extrinsic apoptotic signaling pathway	6.5.E-04
positive regulation of transcription by RNA polymerase II	1.0.E-03
cell-matrix adhesion	1.4.E-03
cell migration	1.5.E-03

Figure EV1. Quantitative PCR for MDAMB231 overexpressing ZCCHC24 and FACS / Western blotting analysis for MDAMB231 or PDX knocked down with siRNA for ZCCHC24.

(**A**) qPCR analysis of MDAMB231 cells overexpressing ZCCHC24 or GFP as controls. Changes in expression were assessed using unpaired *t* tests. (*P* values: *NOTCH2*: 9.7×10^{-4}, *ZEB1*: 0.0023, *CDH11*: 6.68×10^{-5}, JAG1: 9.0×10^{-4}) (***$P < 0.005$, $N = 3$ biological replicates each). (**B**) FACS analysis (CD44 and NRP1) of TNBC cell lines (MDAMB231 and HCC38) or PDX (Patient #1) knocked down with siRNA for ZCCHC24. (**C**) Western blotting analysis for MDAMB231 knocked down with siRNA for ZCCHC24. (**D**) Gene ontology analysis of common differentially expressed genes in the RNA-Seq analysis and destabilized genes in the BRIC-Seq analysis. The specificity of the gene ontology was tested using DAVID software (https://david.ncifcrf.gov/tools.jsp), following the manufacturer's protocol. Data information: Data are presented as mean ± SD (**A**).

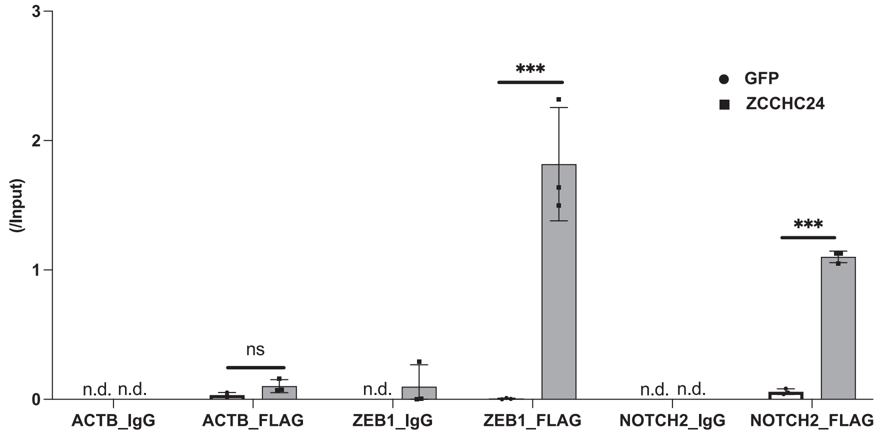

Figure EV2. ZCCHC24 binds to mRNA of genes important for tumor progression and breast cancer stemness.

RIP-qPCR analysis of ZCCHC24. Differences in enrichment were tested using analysis of variance, followed by Tukey's post hoc test. (*P* values: *ACTB* FLAG: 1.0, *ZEB1* FLAG: 1.25×10^{-12}, *NOTCH2* FLAG: 1.11×10^{-7}) ($N = 3$ biological replicates each, $^{***}P < 0.005$, n.d.: not detected, ns: not significant). Data information: Data are presented as mean ± SD.

 © The Author(s)

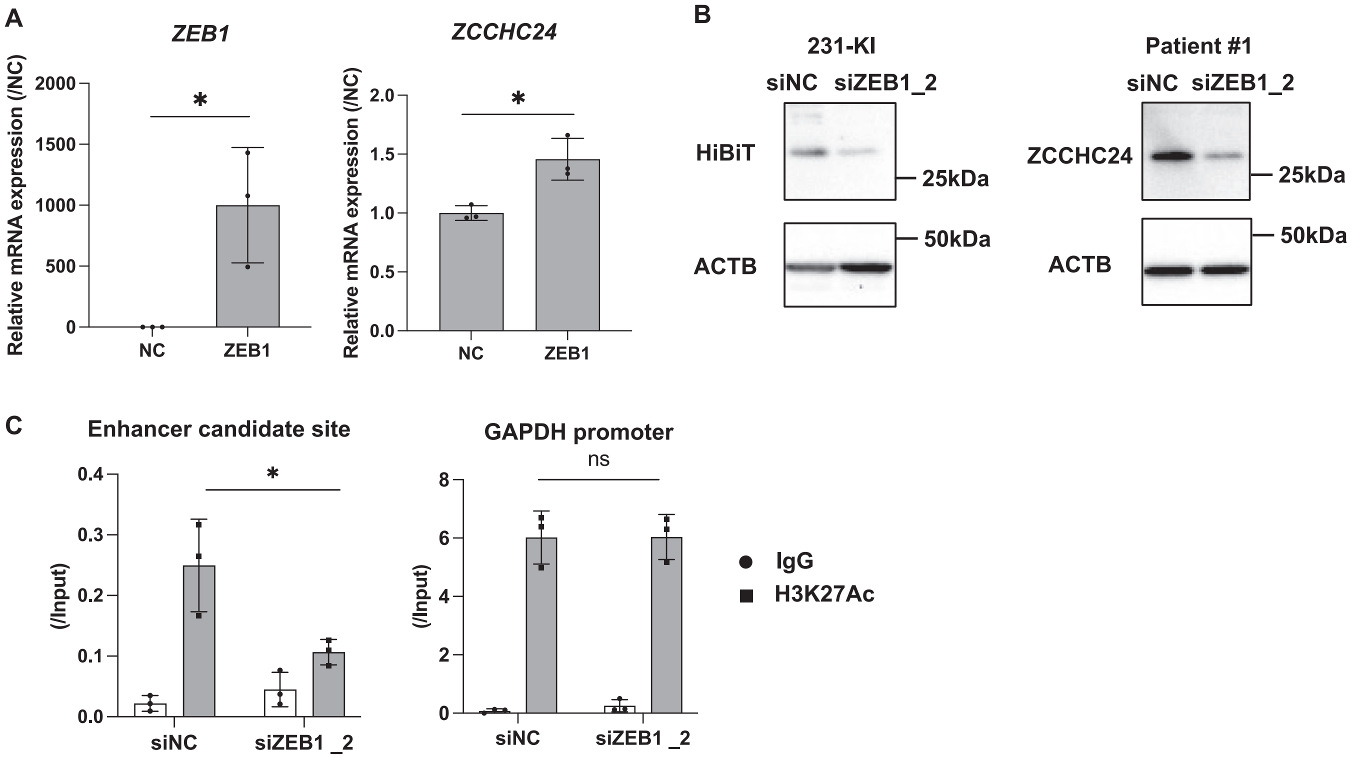

Figure EV3. ZEB1, JUN, and YAP transcriptionally regulate ZCCHC24.

(**A**) qPCR analysis of MDAMB231 overexpressing ZEB1. Changes in gene expression were analyzed using an unpaired t test. (*P* value: *ZEB1*: 0.022, *ZCCHC24*: 0.014) ($N = 3$ biological replicates each, **P* < 0.05). (**B**) Western blot analysis of MDAMB231 and PDX (Patient #1) cells knocked down with siRNA for ZEB1 or the negative control (NC). (**C**) Chromatin immunoprecipitation (ChIP) analysis of MDAMB231 knocked down with siRNA against ZEB1 or negative control (NC). Differences in enrichment were tested using analysis of variance, followed by Tukey's post hoc test. (*P* values: Enhancer candidate site: 0.014, *GAPDH* promoter: 1.0) ($N = 3$ biological replicates each, **P* < 0.05). Data information: Data are presented as mean ± SD (**A**, **C**).

© The Author(s)

A

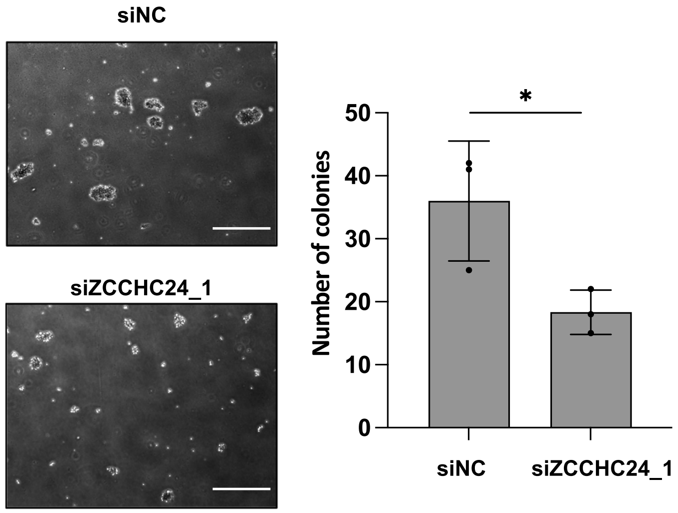

B

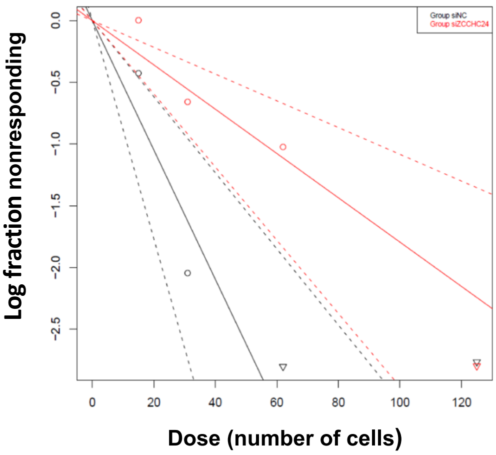

1/(stem cell frequency)	lower	estimate	upper
siNC	32.4	19.1	11.3
siZCCHC24_1	92.3	55.7	33.7

p=0.00493

C

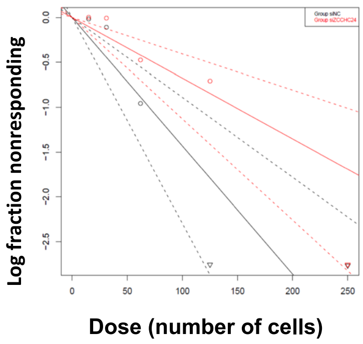

Group	Lower	Estimate	Upper
siNC	112	69.9	43.5
siZCCHC24_1	247	147.8	88.5

p=0.0228

D

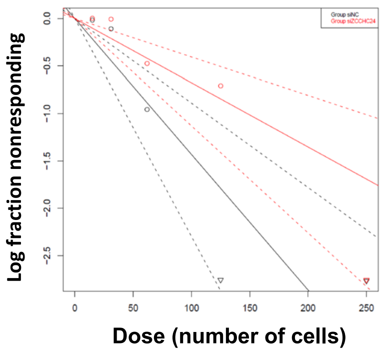

Group	Lower	Estimate	Upper
siNC	112	69.9	43.5
siZCCHC24_1	247	147.8	88.5

p=0.045

E

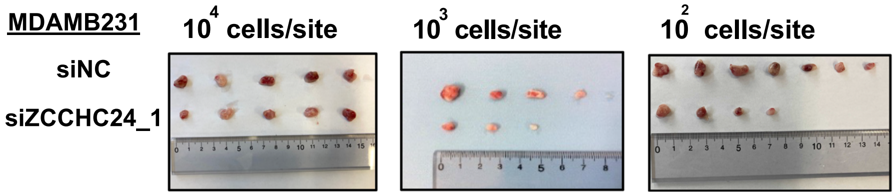

	Cells per site			*p* value vs control	1/(stem cell frequency) (Lower-Upper)
	10^4	10^3	10^2		
siNC	5/5	5/5	7/8		48.6 (117-20.4)
siZCCHC24_1	5/8	4/5	4/8	1.37×10^{-4}	520.5 (1302-208.2)

 © The Author(s)

Figure EV4. ZCCHC24 knockdown downregulates tumor formation in vitro and in vivo.

(**A**) Sphere-formation assay of MDAMB231 cells knocked down with ZCCHC24 siRNA. Differences in the number of spheres formed were tested using unpaired *t* tests. (P value = 0.040) (N = 3 biological replicates each; *$P < 0.05$). (**B**) In vitro extremely limited dilution assay (ELDA) of MDAMB231 knocked down with siRNA for ZCCHC24. Tumor formation ability was tested using the likelihood ratio test of the single-hit model, as shown on the ELDA software website (https://bioinf.wehi.edu.au/software/elda/) by the manufacturer. (P value = 0.00493). (**C**) In vitro extremely limited dilution assay (ELDA) of HCC38 knocked down with siRNA for ZCCHC24. Tumor formation ability was tested using the likelihood ratio test of the single-hit model, as shown on the ELDA software website by the manufacturer. (P value = 0.0228). (**D**) In vitro extremely limited dilution assay (ELDA) of PDX (patient #1) knocked down with siRNA for ZCCHC24. Tumor formation ability was tested using the likelihood ratio test of the single-hit model, as shown on the ELDA software website by the manufacturer. (P value = 0.045). (**E**) Comparison of tumor formation ability in vivo of MDAMB231 knocked down with siRNA for ZCCHC24 or negative control. The cells were transplanted into seven-week-old female nude mice. Tumor formation ability was tested using the likelihood ratio test of the single-hit model, as shown on the manufacturer's ELDA software website (P value: 1.37×10^{-4}). Data information: Data are presented as mean ± SD (**A**). (**A**) Scale bar 50 µm.

© The Author(s)

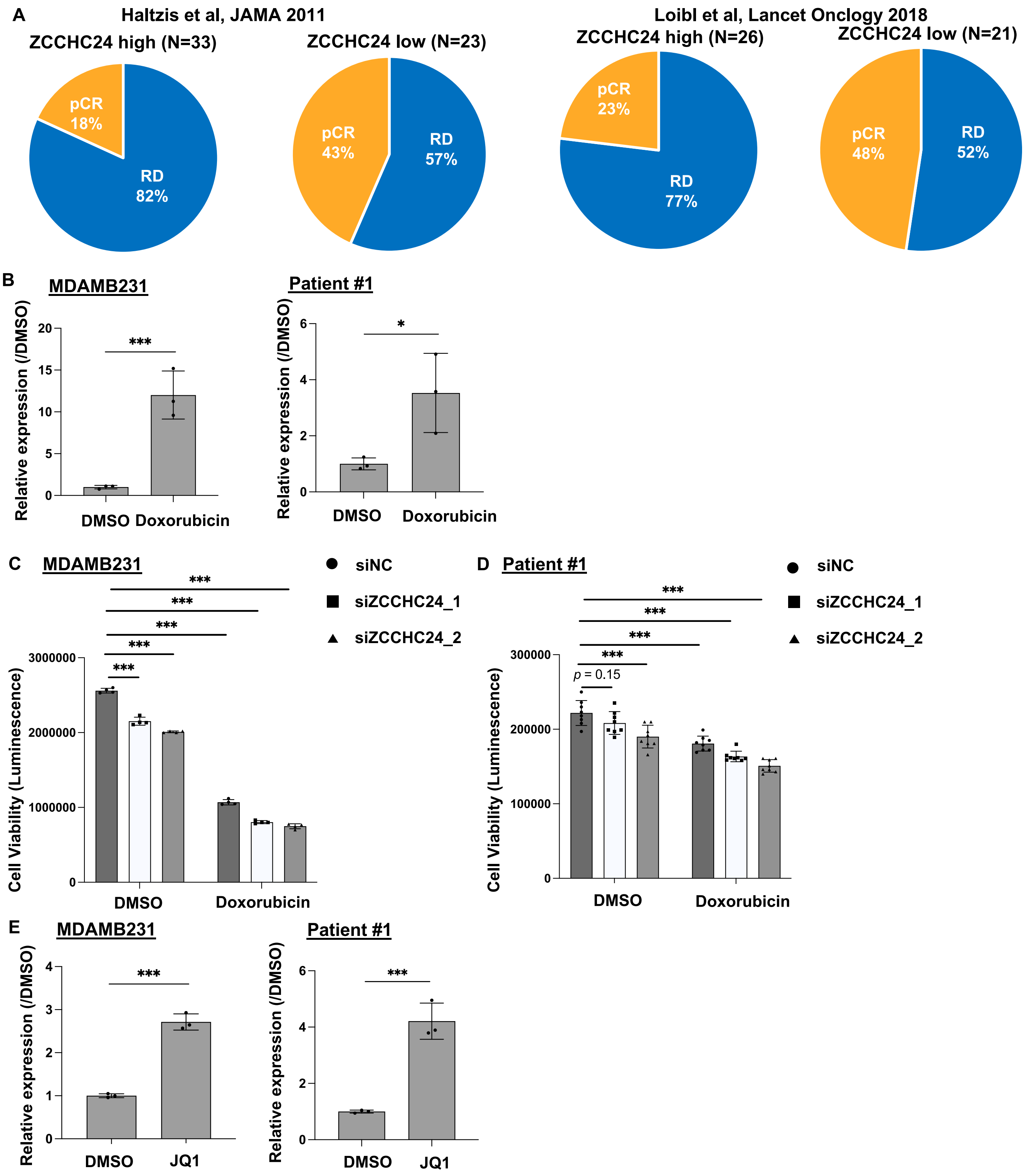
A
Haltzis et al, JAMA 2011
ZCCHC24 high (N=33)
pCR 18%
RD 82%
ZCCHC24 low (N=23)
pCR 43%
RD 57%
Loibl et al, Lancet Onclogy 2018
ZCCHC24 high (N=26)
pCR 23%
RD 77%
ZCCHC24 low (N=21)
pCR 48%
RD 52%
B
MDAMB231
Relative expression (/DMSO)
DMSO Doxorubicin
Patient #1
Relative expression (/DMSO)
DMSO Doxorubicin
C
MDAMB231
Cell Viability (Luminescence)
DMSO Doxorubicin
siNC
siZCCHC24_1
siZCCHC24_2
D
Patient #1
Cell Viability (Luminescence)
p = 0.15
DMSO Doxorubicin
siNC
siZCCHC24_1
siZCCHC24_2
E
MDAMB231
Relative expression (/DMSO)
DMSO JQ1
Patient #1
Relative expression (/DMSO)
DMSO JQ1

 © The Author(s)

Figure EV5. ZCCHC24 expression and clinical prognosis in clinical trials and combined use of a chemotherapy drug and siRNA against ZCCHC24.

(**A**) Clinical prognosis (pathologic complete response (pCR) and residual disease (RD)) and RNA expression of ZCCHC24 in clinical trials (Hatzis et al, 2011; Loibl et al, 2018) with neoadjuvant chemotherapy. (**B**) qPCR analyses of *ZCCHC24* for MDAMB231 or PDX (Patient #1) with the addition of 0.1% DMSO or 1 μM doxorubicin. Changes in expression were assessed using unpaired *t* tests. (*P* values: MDAMB231:0.0027, PDX (Patient #1): 0.037) ($N = 3$ biological replicates each, *$P < 0.05$, ***$P < 0.005$). (**C**) Cell viability assay of MDAMB231 with siRNA transfection and treated with 0.1% DMSO or 500 nM doxorubicin. Differences in cell viability were tested using Dunnett's test (DMSO with siNC as the control). (*P* values: DMSO with siZCCHC24_1: $P < 2.22 \times 10^{-16}$, DMSO with siZCCHC24_2: $P < 2.22 \times 10^{-16}$, doxorubicin with siNC: $P < 2.22 \times 10^{-16}$, doxorubicin with siZCCHC24_1: $P < 2.22 \times 10^{-16}$, doxorubicin with siZCCHC24_2: $P < 2.22 \times 10^{-16}$) ($N = 4$ biological replicates each, ***$P < 0.005$). (**D**) Cell viability assay for PDX (Patient #1) with siRNA transfection and addition of 0.1% DMSO or 1 μM doxorubicin. Differences in cell viability were tested using Dunnett's test. (DMSO with siNC was used as a control). (*P* values: DMSO with siZCCHC24_1: 0.145, DMSO with siZCCHC24_2: 5.61×10^{-5}, doxorubicin with siNC: 9.77×10^{-7}, doxorubicin with siZCCHC24_1: 5.43×10^{-12}, doxorubicin with siZCCHC24_2: 1.67×10^{-14}) ($N = 8$, biological replicates each, ***$P < 0.005$). (**E**) qPCR analyses of *ZCCHC24* for MDAMB231 or PDX (Patient #1) with the addition of 0.1% DMSO or 1 μM JQ1. Changes in expression were assessed using unpaired *t* tests. (*P* alues: MDAMB231: 1.1×10^{-4}, PDX (Patient #1): 9.9×10^{-4}) ($N = 3$ biological replicates each, ***$P < 0.005$). Data information: Data are presented as mean ± SD (**B–E**).

© The Author(s)
